# The informal curriculum of family medicine – what does it entail and how is it taught to residents? A systematic review

**DOI:** 10.1186/s12875-020-01120-1

**Published:** 2020-03-11

**Authors:** Erica Rothlind, Uno Fors, Helena Salminen, Per Wändell, Solvig Ekblad

**Affiliations:** 1 Culture Medicine, Department of Learning, Informatics, Management and Ethics, Karolinska Institutet, Stockholm, Sweden; 2 Department of Computer and Systems Sciences, Stockholm University, Stockholm, Sweden; 3 Department of Neurobiology, Care Sciences and Society, Division of Family Medicine and Primary Care, Karolinska Institutet, Huddinge, Sweden; 4 Academic Primary Health Care Centre, Region Stockholm, Stockholm, Sweden

**Keywords:** Informal/hidden curriculum, Family medicine, General practice, Post-graduate education

## Abstract

**Background:**

The informal curriculum is a seemingly well-explored concept in the realm of medical education. However, it is a concept with multiple definitions and the term “the hidden curriculum” is often used interchangeably. In short, they both refer to the implicit learning taking place outside the formal curriculum, encompassing both a trickling down effect of organizational values and attitudes passed on by a mentor or colleague. While the informal curriculum is a recurrent theme in medical education literature; it is seldom discussed in Family Medicine. As the informal curriculum is likely to be highly influential in the forming of future family practitioners, our aim was to explore the area further, with respect to the following: which elements of the informal curriculum are applicable in a Family Medicine context and what educational interventions for Family Medicine residents, visualizing the various educational elements of it, have been performed?

**Methods:**

We conducted a systematic review comprising iterative literature searches and a narrative synthesis of the results.

**Results:**

Twenty articles, published between 2000 and 2019, were included in the analysis which resulted in three partly interrelated themes comprising the informal curriculum in Family Medicine: gaining cultural competence, achieving medical professionalism and dealing with uncertainty. The themes on cultural competence and uncertainty seemed to be more contextual than professionalism, the latter being discussed in relation to the informal curriculum across other medical disciplines as well. Formalized training for Family Medicine residents in aspects of the informal curriculum appeared to be lacking, and in general, the quality of the few interventional studies found was low.

**Conclusions:**

Important aspects of being a family practitioner, such as cultural competence and dealing with uncertainty, are learned through a context-dependent informal curriculum. In order to ensure a more uniform base for all residents and to reduce the impact of the individual supervisor’s preferences, complementary formalized training would be beneficial. However, to date there are too few studies published to conclude how to best teach the informal curriculum.

**Trial registration:**

The systematic review was registered with Prospero; registration number CRD42018104819.

## Background

In general, post-graduate education for residents in Family Medicine (FM) takes place experientially in the workplace, where learning occurs mainly through practice and interpersonal interactions [1–3]. This type of implicit learning taking place outside the formal learning environment is sometimes referred to as being part of the *informal curriculum* [1, 4, 5], which in literature is often used interchangeably with the term *hidden curriculum* [5]. However, going back to an early definition in relation to medical education, a distinction was made where the hidden curriculum is “a set of influences that function at the level of organizational structure and culture” [1, 6], including, for example, the transferal of customs viewed as common knowledge within the respective field [1]. Whereas the interpersonal form of teaching, in an ad hoc manner, is emphasized in the informal curriculum [1]. Although these categories were originally used to describe undergraduate medical education, they are now widely applied in the context of post-graduate (and workplace) learning as well [1, 7, 8].

For the purpose of this review, we have chosen to incorporate both of these terms, the reason for this being their often ambiguous and interchangeable use in literature [5]. In addition, similar terms, such as *tacit knowledge*, were also included, since our primary focus was to capture and describe what is learnt *outside* the formal curriculum. For readability we have chosen to consequently use the term informal curriculum; meaning that which is not included and taught within a formalized curriculum.

The existence of the informal curriculum is no longer questioned, but in order to understand why and how this is significant, one must recognize that medical education is a socio-cultural process through which the physician’s identity is gradually being shaped [1, 6, 9, 10]. A daily exposure to behaviors in the field of medical practice is likely to influence this process to a greater extent than what is being taught through the formal curriculum in the classroom [11, 12]. There is, however, an ongoing debate on whether it is useful trying to pinpoint what this entails or to define that “which no longer seems to be hidden” [5, 7, 13]. Although the informal curriculum is likely to include common denominators across different fields of medical education, it is also likely to be contextual [5, 14–16]. It has been argued that in order to increase our understanding of the concept, research should explore what the hidden curriculum constitutes in specific areas and levels of education rather than just viewing it as a broad term [5].

The specific area of interest we chose for this review is Family Medicine (FM). Although, being a family doctor is laden with traditional views, FM is a fairly modern specialty; only recently (and still not universally) evolving into a discipline of its own with distinct educational requirements [17]. By default, learning through experience has long since been an established way to learn in the field of FM [3, 18, 19]. As the amount of training gradually becomes more workplace-based throughout a physician’s education, the informal curriculum is likely to have its greatest impact on residents with the absolute majority of their training taking place in the clinic [2, 3, 17]. In a Scandinavian context this is maybe even more relevant since virtually all training is workplace-based [20]. Thus, we theorized that the informal curriculum would be highly relevant for FM residents.

In summary, significant aspects of becoming a family practitioner are likely to be taught within the non-existing framework of the informal curriculum. To understand its impact on FM residents, we aimed to explore what this term entails in the context of FM, and to assess current educational interventions. In order to achieve this, we formulated two review questions:

1. Which elements of the informal curriculum are applicable in a Family Medicine context?
2. What structured educational interventions for Family Medicine residents, visualizing the various elements of the informal curriculum, have been performed? And how are their respective impact and strength of findings rated?

## Methods

A review protocol was registered with Prospero 2018-08-09. The protocol can be accessed through the Prospero website, registration number CRD42018104819.

### Search strategy

An initial literature search was conducted in February 2018, using the following databases: Medline, Epub Ahead of Print, In-Process & Other Non-Indexed Citations, Ovid MEDLINE(R) Daily and Ovid MEDLINE(R) (Ovid), Psycinfo (Ovid), Web of Science Core Collection and ERIC (ProQuest).

The MeSH-terms (Medical Subject Headings) identified for searching Medline (OVID) were adapted under the same principle as the corresponding vocabularies in Psycinfo and ERIC. Throughout the project the search string was iteratively developed as additional themes emerged in the analysis for question 1. Each search concept was complemented with relevant free-text terms like uncertainty, general practitioner, family practitioner, and tacit knowledge. The free-text terms were, if appropriate, truncated and/or combined with proximity operators.

No restrictions to publication types or language were made and the databases were searched from inception. The searches were performed by two librarians at the Karolinska Institutet University Library in February 2018 and a final updated search took place in March 2019. The updated search strings included terms specifically targeting what we identified as parts of the informal curriculum. Reference lists of included papers were also hand searched for relevant articles.

The search strategies are available in Additional files 1, 2, 3 and 4. PRISMA guidelines were adhered to.

### Inclusion criteria

For question 1 (Q1): Articles discussing any aspect of the informal curriculum in relation to FM were included.

For question 2 (Q2): Articles including an educational intervention for residents aimed at addressing the informal curriculum in FM (as identified by analysis of articles found during the initial search string), were included. Articles describing educational interventions aimed at other educational levels (e.g. medical students) or at other health care professions were excluded. Interventional studies involving other primary care physicians or residents from other specialties were included only when the result for FM residents was reported separately. Articles describing a “relevant” curriculum, but which did not present an actual evaluated intervention were also excluded.

Since we anticipated identifying a limited number of publications, no restriction on study type was applied.

### Title and abstract review

After having removed duplicates the first author reviewed the titles and abstracts. If there were no obvious reason to exclude the papers, based on the information in the abstracts, they were included for full-text review. The selected articles were collected in EndNote software.

The same procedure was applied for both Q1 and Q2 in a consecutive manner, with updated literature searches in between (see above).

### Full-text review and data extraction

For Q1 and Q2, all full-text articles were read in whole by the first author and at least one of the co-authors. The articles were evenly distributed among the co-authors. Two data extraction sheets (one for each question respectively) were completed independently by the authors for each article.

In the data extraction sheet for Q1 (see Additional file 5) a free-text field was included where suggested elements of the informal curriculum, covered by the article, were written down and exemplified with quotes.

For Q2 a modified BEME-tool (Best Evidence Medical Education) was used for data extraction as well as quality assessment (see Additional file 6). The BEME-tool was chosen since it is well established in the field of medical education [21]. Fields included (but not limited to) were the following: evaluation methods, expected learning outcome, context (including setting, number of participants and educational intervention), impact of intervention, rating of evaluation methods and strength of findings.

The extracted data was then discussed in consecutive group sessions with the other co-authors, where agreement on which articles to include for each question was reached.

### Data synthesis and analysis

A narrative synthesis was performed [22]. Using the extracted data, a preliminary synthesis of the findings was developed. Relationships within and between studies were explored through analysis of content and theme; the concepts identified where tabulated and after further analysis of their correlation, the overall themes were developed [22]. This iterative part of the analysis did not include any formal quality assessment of the individual papers included in the study, since the aim was to provide a narrative synthesis of what the hidden curriculum in FM contains, according to published literature. The process of developing concepts and themes is outlined in Additional file 7, in order to enhance trustworthiness and decrease the risk of bias.

In addition, for Q2, Kirkpatrick’s hierarchy, being an established model in the field of medical education, was used to assess the impact of the intervention [23]. To assess the strength of the findings presented in the included papers we used the BEME-classification scale as presented in BEME-guide no. 13 [21]. When discrepancies in scoring occurred, the other co-authors read and assessed the article as well in order to reach a consensus.

## Results

**Fig. 1:**
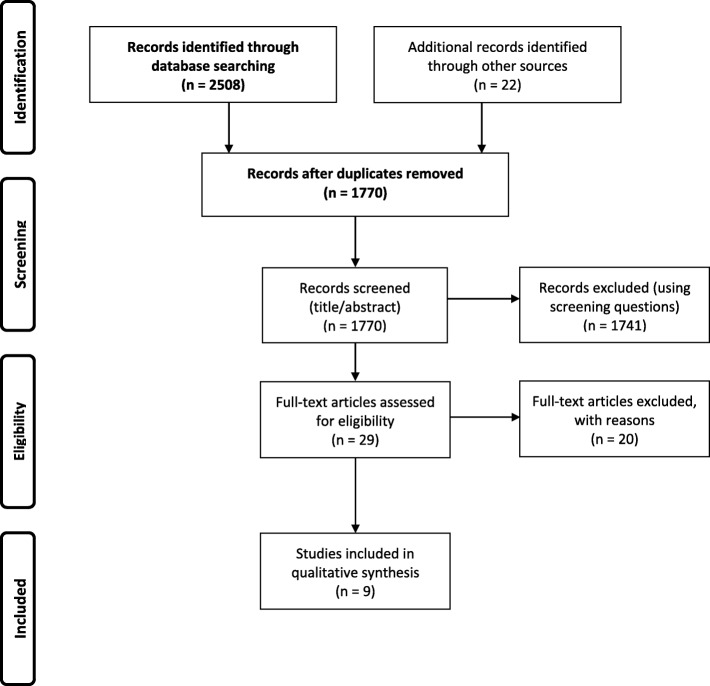
PRISMA flow chart; article selection process for question 1

**Fig. 2:**
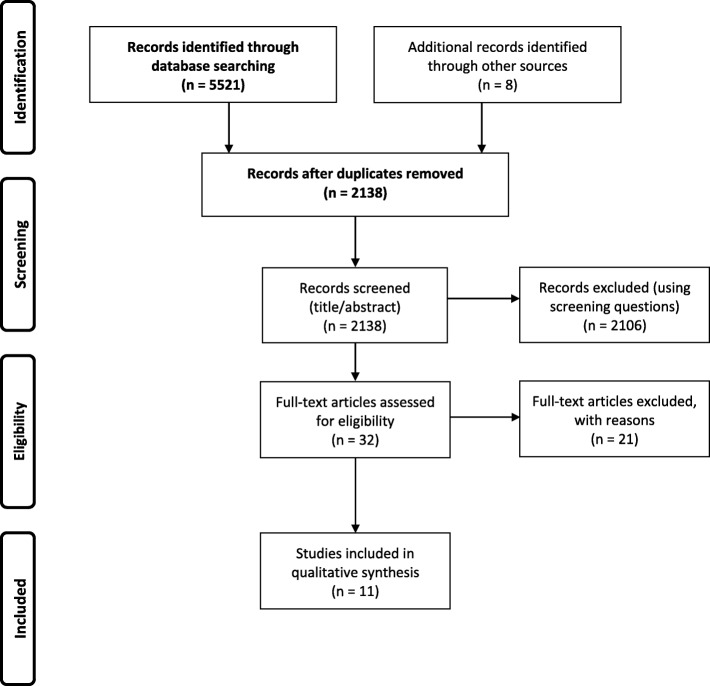
PRISMA flow chart; article selection process for question 2

In our initial search, 1748 articles were identified (after duplicates had been removed) and included in the title and abstract review. After manually searching the reference lists of the articles included in the full-text review, 22 additional articles were added. In total, 29 articles met the inclusion criteria for Q1. The complementary searches, performed after analyzing data for Q1, generated in total 2130 articles (after duplicates had been removed), manual searching generated an additional 8 articles. Of these, 32 articles met the inclusion criteria for Q2. Reviewing the full texts resulted in the final numbers of 9 studies included for Q1 and 11 studies included for Q2. The articles were published between 2000 and 2019. Figures 1 and 2 display the article selection processes in PRISMA flow charts [24]. Key features of the studies included are described in Additional files 5 and 6.

### Elements of the informal curriculum applicable in a family medicine context (Q1)

#### The informal curriculum

The articles studied seemed unified in agreement on a significant existence of an informal curriculum in Family Medicine. However, definitions varied on what this meant. The informal curriculum is clearly defined in only one of the included articles, where it is juxtaposed to the formal curriculum as something that has not been “organized into a coherent whole [ …] written into curricular components” [25]. The hidden curriculum is defined in three articles [26–28], with two of those referring to Hafferty’s established definition of “a set of influences that function at the level of organizational structure and culture” [26, 27] and one article using the following, more prosaic, definition: “a set of values students learn no matter what we decide to teach them” [28]. Explicit definitions are lacking in five of the articles [29–33], but the terms are discussed in relation to interpersonal learning, and as in being a continuous influence reaching beyond medical school [29, 33]. Moreover, related concepts such informal learning, opportunistic learning and ad hoc learning were found; sometimes they were also used interchangeably [30, 31].

Through the process of narrative synthesis three overall, and partly intersecting, elements constituting the informal curriculum of FM were developed: gaining cultural competence, achieving medical professionalism and dealing with uncertainty.

#### Gaining cultural competence

The first element - ‘gaining cultural competence’ - was derived from six of the articles included; there were two surveys, two qualitative studies, one review, and one opinion article dealing with this subject matter [25–28, 30, 31]. Cultural competence is a broad and complex concept and in this review we have chosen to use the most commonly cited definition developed by Cross, et al.: “Cultural and linguistic competence is a set of congruent behaviors, attitudes, and policies that come together in a system, agency, or among professionals that enables effective work in cross-cultural situations” [34]. The concept of cultural humility is also worth mentioning. It is described as a closely related term, albeit, by some, as a separate concept [35]. It incorporates “a lifelong commitment to self-evaluation and critique, to redressing the power imbalances in the physician-patient dynamic, and to developing mutually beneficial and non-paternalistic partnerships with communities on behalf of individuals and defined populations” [35]. For the purpose of this review we have chosen to combine the two definitions under the same theme.

The gaining of cultural competence was, to a great extent, agreed to occur in an informal manner [25–27, 30, 31]. A large survey of FM residency programs in the U.S. also confirmed that, in the majority of the programs included, cultural competence was identified as being part of the informal curriculum [25]. Although experiential learning, through for example exposure to cultural diversity, was discussed as an important way of learning [25–27, 30, 31], the risks of ad hoc learning – having to rely on individual patient encounters as triggers for learning or being over-reliant on a supervisor’s knowledge and interest – were also discussed [27, 30, 31]. A review concluded that a best practice method of ensuring cultural competency training has not yet been established [30]. However, formal education is more likely to improve cultural competency than merely being exposed to culturally diverse patients, even when having access to good role models [30].

#### Achieving medical professionalism

The second element – ‘medical professionalism’ – is also a complex and broad concept varying over time and within cultural contexts, and can be assessed on several levels: individual, inter-personal, and societal-institutional [36]. Professionalism is not only closely associated with a higher quality outcome of health care, but also with increased patient satisfaction, improving physician-patient relationships as well as with a higher career satisfaction [37–39]. Four articles discussing this theme were included; one qualitative study and three opinion articles [28, 29, 32, 33]. The included articles sorted under this term in our review mainly dealt with interpersonal aspects: the ability to engage with and establish a sound relationship with patients, working collaboratively with colleagues, and inter-professionally: avoiding reinforcing hierarchies [28, 29, 32, 33]. Individual aspects such as the importance of self-awareness and reflection were also discussed [32, 33]. Professionalism was discussed as being learned through role models as part of an informal curriculum, where acceptable professional behavior mainly was identified through observation [28, 33].

#### Dealing with uncertainty

The third element – ‘dealing with uncertainty’ – was derived from two of the articles included; one qualitative study and one opinion article [28, 33]. Uncertainty is a concept with a variety of constructs, which is beyond the scope of this article to discuss in depth. However, in summary, it often refers to the diagnostic uncertainty inherent in the nature of the field of medicine and is a product of a combination of clinical judgement, information processing skills and biomedical knowledge [40–42]. This also reflects what was discussed in the articles included in this review where dealing with uncertainty involved dealing with not knowing the answer, as well as dealing with situations when there is no correct answer [28, 43]. It also involved dealing with uncertainty in terms of what could be considered “appropriate” behaviour and in relation to what should be discussed overtly with patients [33].

### Educational interventions for family medicine residents, visualizing the various elements of the informal curriculum (Q2)

#### Design/data collection

Six quantitative studies [43–48] were identified for this review, out of which five described cultural competence interventions [44–48]. Single group pre-post design was used in three of these studies [43, 47, 48]; and one was non-comparative, with surveys administered after the intervention [46]. One study compared the intervention group to a control group, although not randomized, since it involved participants who had applied for a certain program with focus on cultural competence [45]. There was, however, one Randomized Control Trial where data was gathered using a self-assessment tool designed to evaluate cultural competence [44]. The most common method of data collection used was questionnaires constructed in alignment with learning outcomes of the respective intervention [44–46, 48]. The study on uncertainty stood out in a positive way using four different scales with established psychometric properties measuring tolerance of uncertainty and ambiguity [43]. One study also used patient ratings of behavior, as well as observations pre-post the intervention, with focus on cultural competence [47].

Two qualitative studies were also included [17, 49] both evaluating interventions aimed at increasing medical professionalism. Semi-structured interviews were used for collecting data, the analysis was performed using grounded theory [49] and a “modified analytic inductive approach” not explicated further [17].

One of the studies used mixed methods, where a qualitative analysis of reflective texts and photos taken by residents were combined with a questionnaire [50]. Although a small study and thus difficult to draw any conclusions from, the innovative approach of using art as a tool for reflection was valued by the participants [50].

Two of the studies only briefly described their methods, which mainly seemed to involve discussions [51, 52].

#### Context and setting

Of the interventions included, six took place in the U.S. [43–45, 47, 49, 51], two in the U.K. [48, 50], two in Canada [17, 52], and one in Australia [46]. The number of participants were reported for all the studies and ranged from 4 to 1467, with the mean number being 171 and the median 18.

There was an equal distribution between isolated workshops taking place within a short time frame (< 3 months) ranging in number of occasions from 1 to 10 [44, 46, 48, 50–52], and the intervention being integrated long-term with various time intervals over the period of at least 1 year [17, 43, 45, 47, 49].

Various educational settings were used; small-group settings [46, 48–52] and a combination of clinical immersive settings with small-group and/or classroom settings [43, 45, 47] were the most common. One study used an internet-based setting for an interactive case-based course [44].

#### Intervention methods

A wide variety of teaching interventions were used, the most common being small-group discussions [45, 46, 48, 51, 52]. One study also used Balint seminars [53] to teach professionalism [49]. Explicit didactic teaching was less present [17, 45, 47]. Only one study explicitly mentioned mentorship [17]. Notably, there were also several creative and maybe less traditional methods used, such as reflecting on clips from the TV-show “Grey’s Anatomy” to improve professional behavior [51], using photography as a means to self-reflection [50], and discussing poetry in order to gain insight into other cultures [46].

#### Expected learning outcomes

Expected learning outcomes were in most of the studies mentioned briefly or in general terms [17, 45, 46, 51] and only a few studies stated them in further detail [47, 48]. Overall, five studies dealt with topics of medical professionalism [17, 49–52], five studies were aimed at increasing cultural competence [44–48] and one study dealt with improving tolerance of uncertainty [43]. One study on cultural competence focused on treating patients with diabetes type 2 [44], while the other four [45–48] had a wider approach, not focusing on any specific disease.

#### Evaluation outcomes

The authors of this review assessed the impact of the interventions according to Kirkpatrick’s hierarchy (see Table 1). There was only one study where a change in behavior could be observed [47], the remaining studies only dealt with learning in terms of perceived modification of attitudes/perceptions/knowledge or skills [17, 43–46, 48–52]. The mean score was 2, and the median was 2.

**Table 1:** Evaluation of educational interventions aimed at teaching FM residents parts of the informal curriculum

Score	Definition	Number of curricula in included papers (***n*** = 11)
Kirkpatrick’s hierarchy: Impact of intervention studied		
1	Evaluation of participation	0
2	Learning: Modification of attitudes/perceptions/knowledge/skills	10
3	Behavior: Change in behavior as result of learning	1
4	Results: Change in organizational practice/benefits to patients	0
BEME: Strength of findings		
1	No clear conclusions can be drawn. Not significant.	3
2	Results ambiguous, but there appears to be a trend.	3
3	Conclusions can probably be based on the results.	5
4	Results are clear and very likely to be true.	0
5	Results are unequivocal.	0

To assess the quality of the included studies we used the BEME-guide. Results are summarized in Table 1. The mean score was 2, and the median was 2.

All studies reported having an evaluation component, but in some instances it was difficult, as a reader, to assess this independently since it was only described briefly [46, 51, 52]. In these instances, the authors of this review chose to grade the impact according to what the authors of the articles reported, however, we judged the strength of these findings as low.

## Discussion

### Statement of principal findings

To the best of our knowledge, this is the first review attempting to describe the informal curriculum in a FM context, and to explore what attempts have been made at formalizing it in terms of educational interventions for FM residents.

In a FM context we found the informal curriculum to be discussed in relation to three different (but not distinctly separate) themes: gaining cultural competence, achieving medical professionalism, and dealing with uncertainty. In addition to elucidate these three themes, our study had two other main findings. First, unlike professionalism, which seems to be a common theme in the informal curriculum across different disciplines [54–56], both cultural competence and uncertainty seem more limited to FM. Second, given the supposed significance of the informal curriculum, there were surprisingly few studies published within the field of FM in general, in particular when it comes to postgraduate education.

### Findings in relation to previous work

The various elements identified will be discussed in relation to previous work in other specialties and on various educational levels.

The element identified as ‘achieving medical professionalism’ seems to be a recurring theme of the informal curriculum across both educational levels and disciplines [54–59]. Our study confirmed that in FM this is no exception.

More interesting are the other elements which seem to be more specific to FM’s informal curriculum: ‘gaining cultural competence’ and ‘dealing with uncertainty’. Cultural competence and dealing with uncertainty are skills likely to be relevant across the field of medicine but maybe, for the moment, more explicitly discussed and valued in FM. Uncertainty has, for example, been suggested as increasingly prevalent in general practice, as patients are more likely to present with early, and thus more undifferentiated, symptoms and signs of illness than in a hospital setting [40, 60]. In addition, it has been argued that within general practice, medical issues are to a greater extent mixed with relational challenges and a range of non-clinical problems, influencing the symptoms presented and further adding to the presence of uncertainty [3]. FM is also a specialty where building rapport with the patient is highly valued; in order to do so, cultural competence is important [61]. Previous studies in other settings have also identified context- or culture-specific themes in the informal curriculum which support our findings of themes particular to FM. Some examples are the reinforcement of a paternalistic approach towards patients found in an Italian study [14], and “the hidden curriculum of isolation” identified in radiology [62].

In addition to themes in the informal curriculum being identified within various specialties, various articles dealing with the subject matter also discuss modes of *how* it is transferred, i.e. informal learning. Existing literature on how the informal curriculum is taught mainly focuses on a pre-graduate context, while research on how informal learning takes place in the workplace is limited [63]. Given that the education of FM residents to a large extent is workplace-based [2, 3], we have chosen to explore literature on workplace learning for comparison.

In models focusing on workplace learning the concept of informal learning has been elaborated and different types have been outlined: implicit, reactive and deliberate [7, 8]. The level of the individual’s intention to learn constitutes the main difference between the three and seems to be of significance for how well knowledge is retained. The most intentional type of leaning, (where knowledge is also most likely to be well retained), is the so-called deliberate informal learning [7, 8]. Although still work-related, it is a form of learning where goals are formulated and time is set aside for learning [7, 8]; whereas in the reactive form of learning the primary purpose is to manage a work task and learning is at best a byproduct. Predictably, the interventions included in our study with learning goals clearly stated in detail also scored higher in strength of findings [47, 48]. Informal learning within the field of medical education is often described as an overt activity, while the covert aspects of learning, like reflection, is not as present [63]. Satisfyingly, however, we did note that reflection in various forms was explicitly mentioned as a learning activity in several of the included studies [43, 48–52].

Looking across all educational levels, role-modeling/mentorship is repeatedly highlighted as important [9, 56, 62, 63]. Given its supposed significance we found it slightly discouraging that only one of the included studies aiming to teach the informal curriculum applied mentorship as a “learning activity” [17]. Several of the included studies had the intervention longitudinally integrated over a longer period, which would have enabled it to be used more extensively.

Formalizing learning activities does not by default equate effective learning [64]. However, formalizing parts of the informal curriculum would likely facilitate a shift from implicit or reactive informal learning to more deliberate forms, where a modification of skills and behaviors is also more probable to occur [7, 8, 63].

### Limitations

We acknowledge that this literature review may not thoroughly reflect the state of existing curricula for Family Medicine residents on cultural competence, professionalism and uncertainty. Although the literature search was extensive, relevant articles may have been missed as curricular interventions tend to be published within the realms of grey literature. Also, the search terms may not fully cover all aspects of that which is outside of the formal curriculum. In order to try to compensate for this, we performed thorough manual searches, which also generated 30 additional articles. In the end, however, the small number of articles included is a limitation, as it is likely to also affect the depth and breadth of the discussion within the various element identified. This is particularly the case for the element ‘dealing with uncertainty’ which was discussed in only two of the included articles.

In addition, we classified the interventions according to the information provided, which in some articles was very brief and at times lacked clarity. Consequently, our descriptions may in some instances be lacking in terms of capturing the full dimensions of the respective curriculum.

Finally, as we anticipated diversity amongst the study designs and the fact that few studies have been published, we set out to do a narrative synthesis [22] rather than a meta-analysis. We recognize however, that this limits the possibility to draw any firm conclusions on how to best teach the informal curriculum.

### Implications and suggestion for future studies

In our opinion recognizing and pinpointing what the informal curriculum entails in various contexts have three main implications. First, awareness might be raised in supervisors of the areas in which they are likely to have a great impact on their respective resident(s). Second, the gaps where there might be a need for formalized learning activities could also be identified. Finally, it would also signal to residents that these aspects of learning to be a family practitioner are valued. In order to further analyze the concept of the informal curriculum in various contexts, using an inductive approach would be beneficial. We would therefore suggest further qualitative studies of the concept in various fields to reduce the risk of continuous generalizations. In addition, by exploring the individual elements inductively, in particular ‘dealing with uncertainty’ where studies seem to be lacking, a more profound understanding of the contextual use of the concepts may be reached.

## Conclusion

The particularities of FM, such as learning to manage a great deal of uncertainty, are to some extent reflected in its informal curriculum; emphasizing the context-dependent nature of the concept. Recognizing the informal curriculum as consisting of multiple informal curricula would likely facilitate a more pragmatic use of the concept in general. In FM in particular, where important aspects of being a family practitioner are learned through the informal curriculum, it would be beneficial to formalize parts of it for two reasons. First it would reduce the dependency on the individual supervisor’s interests or preferences by providing a more uniform base for all residents. Second, formalizing parts of the training might facilitate learning which is more likely to be retained; and be reflected in changes in attitudes and behaviors. The current literature is, however, not sufficient enough to conclude how to best teach the informal curriculum.

## Supplementary information

**Additional file 1.** Original search string. Search strings used for the various data bases searched.

**Additional file 2.** Updated search string including uncertainty. Updated search strings including “uncertainty” and similar terms.

**Additional file 3.** Updated search string including professionalism. Updated search strings including “medical professionalism” and similar terms.

**Additional file 4.** Updated search string including cultural competence. Updated search strings including “cultural competence” and similar terms.

**Additional file 5.** A summary of the studies included for question 1. Articles included are listed and described using the following headings: “defines informal curriculum”, “type of article”, “participants”, and “elements of the informal curriculum”.

**Additional file 6.** A summary of the studies included for question 2. Articles included are listed and described using the following headings: “informal curriculum theme”, “evaluation methods”, “expected learning outcome”, “context”, “impact of intervention”, and “strength of findings”.

**Additional file 7.** A summary of the development of concepts and themes from articles included for question 1. Summarizes data underlying the development of the concepts and themes for question 1.

## Data Availability

As the results of the synthesis are fully referenced within the article, additional data and materials are not available.

## Abbreviations

BEME: Best evidence medical education
FM: Family medicine
