# Supplementary material for: The informal curriculum of family medicine – what does it entail and how is it taught to residents? A systematic review
Source: BMC Fam Pract. 2020 Mar 11;21:49. doi: 10.1186/s12875-020-01120-1 (PMC7066821; doi:10.1186/s12875-020-01120-1)
Supplement: Supplementary file 2 — Additional file 2. Updated search string including uncertainty. Updated search strings including “uncertainty” and similar terms. [file 12875_2020_1120_MOESM2_ESM.docx]

**Additional file 2. Updated search string including uncertainty**

1. Medline

| Interface: Ovid  Date of Search: 5 March 2019  Number of hits: 1137  Comment: In Ovid, two or more words are automatically searched as phrases; i.e. no quotation marks are needed | Field labels   - exp/ = exploded MeSH term - / = non exploded MeSH term - .ti,ab,kf. = title, abstract and author keywords - adjx = within x words, regardless of order - * = truncation of word for alternate endings - ? = zero or one character |
| --- | --- |
| 1. ((uncertain* or undefined or medically unexplain* or ambigu* or vocational threshold* or (complex* adj1 (patient* or diagnos* or clinical*))) adj5 (education* or teaching or learning or elearning or training or curriculum or tacit knowledge or student or intern? or internship* or resident? or residency or competen* or skill?)).ti,ab,kf.  2. Physicians/  3. General Practitioners/  4. Physicians, Family/  5. Physicians, Primary Care/  6. Physician's Role/  7. Physician-Patient Relations/  8. exp General Practice/  9. (doctor* or physician* or general practi* or gp or gps or family practi* or primary health care or primary healthcare or primary care or registrar*).ti,ab,kf.  10. or/2-9    11. 1 and 10  12. ((uncertain* or undefined or medically unexplain* or ambigu* or vocational threshold* or (complex* adj1 (patient* or diagnos* or clinical*))) adj5 (doctor* or physician* or general practi* or gp or gps or family practi* or primary health care or primary healthcare or primary care or registrar*)).ti,ab,kf.  13. exp Education, Medical/  14. Education/  15. education.fs.  16. exp Teaching/  17. exp Learning/  18. exp Curriculum/  19. exp Inservice Training/  20. Clinical Competence/  21. Cultural Competency/  22. Culturally Competent Care/  23. (education* or teaching or learning or elearning or training or curriculum or tacit knowledge or student or intern? or internship* or resident? or residency).ti,ab,kf.  24. ((staff or professional or workforce or work force) adj3 development).ti,af,kf.  25. (competen* or skill?).ti,ab,kf.  26. or/13-25    27. 12 and 26  28. *Uncertainty/  29. *Medically Unexplained Symptoms/  30. *Somatoform Disorders/  31. (uncertain* or undefined or medically unexplain* or ambigu* or vocational threshold* or (complex* adj1 (patient* or diagnos* or clinical*))).ti.  32. or/28-31  33. 10 and 26 and 32  34. 11 or 27 or 33 | |

2. Web of Science Core Collection

| Interface: Clarivate Analytics  Date of Search: 5 March 2019  Number of hits: 1113 | Field labels   - TS/Topic = title, abstract, author keywords and Keywords Plus - NEAR/x = within x words, regardless of order - * = truncation of word for alternate endings - $ = zero or one character |
| --- | --- |
| #1 **TOPIC:** (((uncertain* or undefined or "medically unexplain*" or ambigu* or "vocational threshold*" or (complex* NEAR/1 (patient* or diagnos* or clinical*))) NEAR/5 (education* or teaching or learning or elearning or training or curriculum or "tacit knowledge" or student or intern$ or internship* or resident$ or residency or competen* or skill$)))  #2 **TOPIC:** ((doctor* or physician* or "general practi*" or gp or gps or "family practi*" or "primary health care" or "primary healthcare" or "primary care" or registrar*))  #3 #2 AND #1  #4 **TOPIC:** (((uncertain* or undefined or "medically unexplain*" or ambigu* or "vocational threshold*" or (complex* NEAR/1 (patient* or diagnos* or clinical*))) NEAR/5 (doctor* or physician* or "general practi*" or gp or gps or "family practi*" or "primary health care" or "primary healthcare" or "primary care" or registrar*)))  #5 **TOPIC:** ((education* or teaching or learning or elearning or training or curriculum or "tacit knowledge" or student or intern$ or internship* or resident$ or residency)) *OR* **TOPIC:** (((staff or professional or workforce or "work force") NEAR/3 development)) *OR* **TOPIC:** ((competen* or skill$))  #6 #5 AND #4  #7 **TITLE:** ((uncertain* or undefined or "medically unexplain*" or ambigu* or "vocational threshold*" or (complex* NEAR/1 (patient* or diagnos* or clinical*))))  #8 #7 AND #5 AND #2  #9 #8 OR #6 OR #3 | |

3. Psycinfo

| Interface: Ovid  Date of Search: 5 March 2019  Number of hits: 442  Comment: In Ovid, two or more words are automatically searched as phrases; i.e. no quotation marks are needed | Field labels   - exp/ = exploded controlled term - / = non exploded controlled term - .ti,ab,id. = title, abstract and author keywords - adjx = within x words, regardless of order - * = truncation of word for alternate endings - ? = zero or one character |
| --- | --- |
| 1. ((uncertain* or undefined or medically unexplain* or ambigu* or vocational threshold* or (complex* adj1 (patient* or diagnos* or clinical*))) adj5 (education* or teaching or learning or elearning or training or curriculum or tacit knowledge or student or intern? or internship* or resident? or residency or competen* or skill?)).ti,ab,id.  2. physicians/  3. general practitioners/  4. family physicians/  5. (doctor* or physician* or general practi* or gp or gps or family practi* or primary health care or primary healthcare or primary care or registrar*).ti,ab,id.  6. or/2-5  7. 1 and 6  8. ((uncertain* or undefined or medically unexplain* or ambigu* or vocational threshold* or (complex* adj1 (patient* or diagnos* or clinical*))) adj5 (doctor* or physician* or general practi* or gp or gps or family practi* or primary health care or primary healthcare or primary care or registrar*)).ti,ab,id.  9. medical education/  10. education/  11. medical internship/  12. medical residency/  13. teaching/  14. exp teaching methods/  15. exp learning/  16. curriculum/  17. continuing education/  18. personnel training/  19. on the job training/  20. inservice training/  21. exp competence/  22. cultural sensitivity/  23. cross cultural communication/  24. exp cross cultural treatment/  25. (education* or teaching or learning or elearning or training or curriculum or tacit knowledge or student or intern? or internship* or resident? or residency).ti,ab,id.  26. ((staff or professional or workforce or work force) adj3 development).ti,af,id.  27. (competen* or skill?).ti,ab,id.  28. or/9-27  29. 8 and 28  30. exp *uncertainty/  31. somatoform disorders/  32. (uncertain* or undefined or medically unexplain* or ambigu* or vocational threshold* or (complex* adj1 (patient* or diagnos* or clinical*))).ti.  33. or/30-32  34. 6 and 28 and 33  35. 7 or 29 or 34 | |

4. ERIC

| Interface: ProQuest  Date of Search: 5 March 2019  Number of hits: 76 | Field labels   - MAINSUBJECT.EXACT.EXPLODE = exploded controlled term - MAINSUBJECT.EXACT = non exploded controlled term - ti = title - ab = abstract - NEAR/x = within x words, regardless of order - * = truncation of word for alternate endings |
| --- | --- |
| ((ti(((uncertain* OR undefined OR "medically unexplain*" OR ambigu* OR "vocational threshold*" OR (complex* NEAR/1 (patient* OR diagnos* OR clinical*))) NEAR/5 (education* OR teaching OR learning OR elearning OR training OR curriculum OR "tacit knowledge" OR student OR intern OR interns OR internship* OR resident OR residents OR residency OR competen* OR skill OR skills))) OR ab(((uncertain* OR undefined OR "medically unexplain*" OR ambigu* OR "vocational threshold*" OR (complex* NEAR/1 (patient* OR diagnos* OR clinical*))) NEAR/5 (education* OR teaching OR learning OR elearning OR training OR curriculum OR "tacit knowledge" OR student OR intern OR interns OR internship* OR resident OR residents OR residency OR competen* OR skill OR skills)))) AND ((MAINSUBJECT.EXACT("Physicians") OR MAINSUBJECT.EXACT("Physician Patient Relationship") OR MAINSUBJECT.EXACT("Family Practice (Medicine)")) OR (ti((doctor* OR physician* OR "general practi*" OR gp OR gps OR "family practi*" OR "primary health care" OR "primary healthcare" OR "primary care" OR registrar*)) OR ab((doctor* OR physician* OR "general practi*" OR gp OR gps OR "family practi*" OR "primary health care" OR "primary healthcare" OR "primary care" OR registrar*))))) OR ((ti(((uncertain* OR undefined OR "medically unexplain*" OR ambigu* OR "vocational threshold*" OR (complex* NEAR/1 (patient* OR diagnos* OR clinical*))) NEAR/5 (doctor* OR physician* OR "general practi*" OR gp OR gps OR "family practi*" OR "primary health care" OR "primary healthcare" OR "primary care" OR registrar*))) OR ab(((uncertain* OR undefined OR "medically unexplain*" OR ambigu* OR "vocational threshold*" OR (complex* NEAR/1 (patient* OR diagnos* OR clinical*))) NEAR/5 (doctor* OR physician* OR "general practi*" OR gp OR gps OR "family practi*" OR "primary health care" OR "primary healthcare" OR "primary care" OR registrar*)))) AND ((MAINSUBJECT.EXACT("Medical Education") OR MAINSUBJECT.EXACT("Graduate Medical Education") OR MAINSUBJECT.EXACT.EXPLODE("Education") OR MAINSUBJECT.EXACT("Professional Education") OR MAINSUBJECT.EXACT.EXPLODE("Educational Methods") OR MAINSUBJECT.EXACT("Teaching") OR MAINSUBJECT.EXACT.EXPLODE("Learning") OR MAINSUBJECT.EXACT.EXPLODE("Curriculum") OR MAINSUBJECT.EXACT("Hidden Curriculum") OR MAINSUBJECT.EXACT("Informal Education") OR **MAINSUBJECT.EXACT("Competence") OR MAINSUBJECT.EXACT("Cultural Literacy")**) OR MAINSUBJECT.EXACT("Teaching (Occupation)") OR (ti(((education* OR teaching OR learning OR elearning OR training OR curriculum OR "tacit knowledge" OR student OR intern OR interns OR internship* OR resident OR residents OR residency) OR ((staff OR professional OR workforce OR "work force") NEAR/3 development) OR (competen* OR skill OR skills))) OR ab(((education* OR teaching OR learning OR elearning OR training OR curriculum OR "tacit knowledge" OR student OR intern OR interns OR internship* OR resident OR residents OR residency) OR ((staff OR professional OR workforce OR "work force") NEAR/3 development) OR (competen* OR skill OR skills)))))) OR (((MAINSUBJECT.EXACT("Physicians") OR MAINSUBJECT.EXACT("Physician Patient Relationship") OR MAINSUBJECT.EXACT("Family Practice (Medicine)")) OR (ti((doctor* OR physician* OR "general practi*" OR gp OR gps OR "family practi*" OR "primary health care" OR "primary healthcare" OR "primary care" OR registrar*)) OR ab((doctor* OR physician* OR "general practi*" OR gp OR gps OR "family practi*" OR "primary health care" OR "primary healthcare" OR "primary care" OR registrar*)))) AND ((MAINSUBJECT.EXACT("Medical Education") OR MAINSUBJECT.EXACT("Graduate Medical Education") OR MAINSUBJECT.EXACT.EXPLODE("Education") OR MAINSUBJECT.EXACT("Professional Education") OR MAINSUBJECT.EXACT.EXPLODE("Educational Methods") OR MAINSUBJECT.EXACT("Teaching") OR MAINSUBJECT.EXACT.EXPLODE("Learning") OR MAINSUBJECT.EXACT.EXPLODE("Curriculum") OR MAINSUBJECT.EXACT("Hidden Curriculum") OR MAINSUBJECT.EXACT("Informal Education") OR **MAINSUBJECT.EXACT("Competence") OR MAINSUBJECT.EXACT("Cultural Literacy")**) OR MAINSUBJECT.EXACT("Teaching (Occupation)") OR (ti(((education* OR teaching OR learning OR elearning OR training OR curriculum OR "tacit knowledge" OR student OR intern OR interns OR internship* OR resident OR residents OR residency) OR ((staff OR professional OR workforce OR "work force") NEAR/3 development) OR (competen* OR skill OR skills))) OR ab(((education* OR teaching OR learning OR elearning OR training OR curriculum OR "tacit knowledge" OR student OR intern OR interns OR internship* OR resident OR residents OR residency) OR ((staff OR professional OR workforce OR "work force") NEAR/3 development) OR (competen* OR skill OR skills))))) AND ti((uncertain* OR undefined OR "medically unexplain*" OR ambigu* OR "vocational threshold*" OR (complex* NEAR/1 (patient* OR diagnos* OR clinical*))))) | |
