# Supplementary material for: The informal curriculum of family medicine – what does it entail and how is it taught to residents? A systematic review
Source: BMC Fam Pract. 2020 Mar 11;21:49. doi: 10.1186/s12875-020-01120-1 (PMC7066821; doi:10.1186/s12875-020-01120-1)
Supplement: Supplementary file 3 — Additional file 3. Updated search string including professionalism. Updated search strings including “medical professionalism” and similar terms. [file 12875_2020_1120_MOESM3_ESM.docx]

**Additional file 3. Updated search string including professionalism**

1. Medline

| Interface: Ovid  Date of Search: 6 March 2019  Number of hits: 688  Comment: In Ovid, two or more words are automatically searched as phrases; i.e. no quotation marks are needed | Field labels   - exp/ = exploded MeSH term - / = non exploded MeSH term - .ti,ab,kf. = title, abstract and author keywords - adjx = within x words, regardless of order - * = truncation of word for alternate endings - ? = zero or one character |
| --- | --- |
| 1. ((professionalism or ethic* or (professional adj3 (development or identity or role))) adj3 (education* or teaching or learning or elearning or training or curriculum or tacit knowledge or intervention* or program*)).ti,ab,kf.  2. General Practitioners/  3. Physicians, Family/  4. Physicians, Primary Care/  5. exp General Practice/  6. Primary Health Care/  7. (general practi* or gp or gps or family practi* or primary health care or primary healthcare or primary care).ti,ab,kf.  8. or/2-7  9. 1 and 8    10. exp Education, Medical, Graduate/  11. Education, Medical, Continuing/  12. Education/  13. education.fs.  14. exp Teaching/  15. exp Learning/  16. exp Curriculum/  17. exp Inservice Training/  18. (education* or teaching or learning or elearning or training or curriculum or tacit knowledge or intervention* or program*).ti,ab,kf.  19. or/10-18  20. *Ethics/  21. *Ethics, Professional/  22. *Ethics, Clinical/  23. *Ethics, Medical/  24. *Professionalism/  25. (ethic* or professionalism or (professional adj3 (development or identity or role))).ti.  26. or/20-25  27. 8 and 19 and 26  28. 9 or 27 | |

2. Web of Science Core Collection

| Interface: Clarivate Analytics  Date of Search: 6 March 2019  Number of hits: 611 | Field labels   - TS/Topic = title, abstract, author keywords and Keywords Plus - NEAR/x = within x words, regardless of order - * = truncation of word for alternate endings - $ = zero or one character |
| --- | --- |
| #1 **TOPIC:** (((professionalism or ethic* or ((professional) NEAR/3 (development or identity or role))) NEAR/3 (education* or teaching or learning or elearning or training or curriculum or "tacit knowledge" or intervention* or program*)))  #2 **TOPIC:** ("general practi*" or gp or gps or "family practi*" or "primary health care" or "primary healthcare" or "primary care")  #3 #2 AND #1  #4 **TOPIC:** (education* or teaching or learning or elearning or training or curriculum or "tacit knowledge" or intervention* or program*)  #5 **TITLE:** (ethic* or professionalism or (professional NEAR/3 (development or identity or role)))  #6 #5 AND #4 AND #2  #7 #6 OR #3 | |

3. Psycinfo

| Interface: Ovid  Date of Search: 6 March 2019  Number of hits: 255  Comment: In Ovid, two or more words are automatically searched as phrases; i.e. no quotation marks are needed | Field labels   - exp/ = exploded controlled term - / = non exploded controlled term - .ti,ab,id. = title, abstract and author keywords - adjx = within x words, regardless of order - * = truncation of word for alternate endings - ? = zero or one character |
| --- | --- |
| 1. ((professionalism or ethic* or (professional adj3 (development or identity or role))) adj3 (education* or teaching or learning or elearning or training or curriculum or tacit knowledge or intervention* or program*)).ti,ab,id.  2. general practitioners/  3. family physicians/  4. (general practi* or gp or gps or family practi* or primary health care or primary healthcare or primary care).ti,ab,id.  5. or/2-4  6. 1 and 5  7. medical education/  8. education/  9. medical internship/  10. medical residency/  11. teaching/  12. exp teaching methods/  13. exp learning/  14. curriculum/  15. continuing education/  16. personnel training/  17. on the job training/  18. inservice training/  19. (education* or teaching or learning or elearning or training or curriculum or tacit knowledge or intervention* or program*).ti,ab,id.  20. or/7-19    21. *ethics/  22. *professional ethics/  23. *bioethics/  24. *professionalism/  25. *professional identity/  26. (ethic* or professionalism or (professional adj3 (development or identity or role))).ti.  27. or/21-26  28. 5 and 20 and 27  29. 6 or 28 | |

4. ERIC

| Interface: ProQuest  Date of Search: 6 March 2019  Number of hits: 126 | Field labels   - MAINSUBJECT.EXACT.EXPLODE = exploded controlled term - MAINSUBJECT.EXACT = non exploded controlled term - ti = title - ab = abstract - NEAR/x = within x words, regardless of order - * = truncation of word for alternate endings |
| --- | --- |
| ((ti(((professionalism OR ethic* OR (professional NEAR/3 (development OR identity OR role))) NEAR/3 (education* OR teaching OR learning OR elearning OR training OR curriculum OR "tacit knowledge" OR intervention* OR program*))) OR ab(((professionalism OR ethic* OR (professional NEAR/3 (development OR identity OR role))) NEAR/3 (education* OR teaching OR learning OR elearning OR training OR curriculum OR "tacit knowledge" OR intervention* OR program*)))) AND (MAINSUBJECT.EXACT("Physicians") OR MAINSUBJECT.EXACT("Family Practice (Medicine)") OR ti("general practi*" OR gp OR gps OR "family practi*" OR "primary health care" OR "primary healthcare" OR "primary care") OR ab("general practi*" OR gp OR gps OR "family practi*" OR "primary health care" OR "primary healthcare" OR "primary care"))) OR ((MAINSUBJECT.EXACT("Physicians") OR MAINSUBJECT.EXACT("Family Practice (Medicine)") OR ti("general practi*" OR gp OR gps OR "family practi*" OR "primary health care" OR "primary healthcare" OR "primary care") OR ab("general practi*" OR gp OR gps OR "family practi*" OR "primary health care" OR "primary healthcare" OR "primary care")) AND (MAINSUBJECT.EXACT("Graduate Medical Education") OR MAINSUBJECT.EXACT("Education") OR MAINSUBJECT.EXACT.EXPLODE("Educational Methods") OR MAINSUBJECT.EXACT("Teaching") OR MAINSUBJECT.EXACT.EXPLODE("Learning") OR MAINSUBJECT.EXACT.EXPLODE("Curriculum") OR MAINSUBJECT.EXACT("Hidden Curriculum") OR MAINSUBJECT.EXACT("Informal Education") OR ti(education* OR teaching OR learning OR elearning OR training OR curriculum OR "tacit knowledge" OR intervention* OR program*) OR ab(education* OR teaching OR learning OR elearning OR training OR curriculum OR "tacit knowledge" OR intervention* OR program*)) AND (MJMAINSUBJECT.EXACT("Ethics") OR MJMAINSUBJECT.EXACT("Professionalism") OR MJMAINSUBJECT.EXACT("Professional Identity") OR ti(ethic* or professionalism or (professional NEAR/3 (development or identity or role))))) | |
