# Supplementary material for: The informal curriculum of family medicine – what does it entail and how is it taught to residents? A systematic review
Source: BMC Fam Pract. 2020 Mar 11;21:49. doi: 10.1186/s12875-020-01120-1 (PMC7066821; doi:10.1186/s12875-020-01120-1)
Supplement: Supplementary file 4 — Additional file 4. Updated search string including cultural competence. Updated search strings including “cultural competence” and similar terms. [file 12875_2020_1120_MOESM4_ESM.docx]

**Additional file 4. Updated search string including cultural competence**

1. Medline

| Interface: Ovid  Date of Search: 29 March 2019  Number of hits: 435  Comment: In Ovid, two or more words are automatically searched as phrases; i.e. no quotation marks are needed | Field labels   - exp/ = exploded MeSH term - / = non exploded MeSH term - .ti,ab,kf. = title, abstract and author keywords - adjx = within x words, regardless of order - * = truncation of word for alternate endings - ? = zero or one character |
| --- | --- |
| 1. ((cultur* or crosscultur* or intercultur* or transcultur* or multicultur*) adj3 (competenc* or understanding or knowledg* or expertise or skill* or sensitiv* or aware* or appropriate* or acceptab* or humility or service* or communicat* or barrier* or divers* or comparison* or identity or specific or background* or value* or belief* or literacy or respect or framework) adj3 (education* or teaching or learning or elearning or training or curriculum or tacit knowledge or intervention* or program*)).ti,ab,kf.  2. General Practitioners/  3. Physicians, Family/  4. Physicians, Primary Care/  5. exp General Practice/  6. Primary Health Care/  7. (general practi* or gp or gps or family practi* or primary health care or primary healthcare or primary care).ti,ab,kf.  8. or/2-7    9. 1 and 8  10. exp Education, Medical, Graduate/  11. Education, Medical, Continuing/  12. Education/  13. education.fs.  14. exp Teaching/  15. exp Learning/  16. exp Curriculum/  17. exp Inservice Training/  18. (education* or teaching or learning or elearning or training or curriculum or tacit knowledge or intervention* or program*).ti,ab,kf.  19. or/10-18  20. *Cultural Competency/  21. *Culturally Competent Care/  22. ((cultur* or crosscultur* or intercultur* or transcultur* or multicultur*) adj3 (competenc* or understanding or knowledg* or expertise or skill* or sensitiv* or aware* or appropriate* or acceptab* or humility or service* or communicat* or barrier* or divers* or comparison* or identity or specific or background* or value* or belief* or literacy or respect or framework)).ti.  23. or/20-22  24. 8 and 19 and 23  25. 9 or 24 | |

2. Web of Science Core Collection

| Interface: Clarivate Analytics  Date of Search: 29 March 2019  Number of hits: 413 | Field labels   - TS/Topic = title, abstract, author keywords and Keywords Plus - NEAR/x = within x words, regardless of order - * = truncation of word for alternate endings - $ = zero or one character |
| --- | --- |
| #1 **TOPIC:** (((cultur* or crosscultur* or intercultur* or transcultur* or multicultur*) NEAR/3 (competenc* or understanding or knowledg* or expertise or skill* or sensitiv* or aware* or appropriate* or acceptab* or humility or service* or communicat* or barrier* or divers* or comparison* or identity or specific or background* or value* or belief* or literacy or respect or framework)) NEAR/3 (education* or teaching or learning or elearning or training or curriculum or "tacit knowledge" or intervention* or program*))  #2 **TOPIC:** ("general practi*" or gp or gps or "family practi*" or "primary health care" or "primary healthcare" or "primary care")  #3 #2 AND #1  #4 **TOPIC:** (education* or teaching or learning or elearning or training or curriculum or "tacit knowledge" or intervention* or program*)  #5 **TITLE:** ((cultur* or crosscultur* or intercultur* or transcultur* or multicultur*) NEAR/3 (competenc* or understanding or knowledg* or expertise or skill* or sensitiv* or aware* or appropriate* or acceptab* or humility or service* or communicat* or barrier* or divers* or comparison* or identity or specific or background* or value* or belief* or literacy or respect or framework))  #6 #5 AND #4 AND #2  #7 #6 OR #3 | |

3. Psycinfo

| Interface: Ovid  Date of Search: 29 March 2019  Number of hits: 197  Comment: In Ovid, two or more words are automatically searched as phrases; i.e. no quotation marks are needed | Field labels   - exp/ = exploded controlled term - / = non exploded controlled term - .ti,ab,id. = title, abstract and author keywords - adjx = within x words, regardless of order - * = truncation of word for alternate endings - ? = zero or one character |
| --- | --- |
| 1. ((cultur* or crosscultur* or intercultur* or transcultur* or multicultur*) adj3 (competenc* or understanding or knowledg* or expertise or skill* or sensitiv* or aware* or appropriate* or acceptab* or humility or service* or communicat* or barrier* or divers* or comparison* or identity or specific or background* or value* or belief* or literacy or respect or framework) adj3 (education* or teaching or learning or elearning or training or curriculum or tacit knowledge or intervention* or program*)).ti,ab,id.  2. general practitioners/  3. family physicians/  4. (general practi* or gp or gps or family practi* or primary health care or primary healthcare or primary care).ti,ab,id.  5. or/2-4  6. 1 and 5  7. medical education/  8. education/  9. medical internship/  10. medical residency/  11. teaching/  12. exp teaching methods/  13. exp learning/  14. curriculum/  15. continuing education/  16. personnel training/  17. on the job training/  18. inservice training/  19. (education* or teaching or learning or elearning or training or curriculum or tacit knowledge or intervention* or program*).ti,ab,id.  20. or/7-19  21. *cultural sensitivity/  22. *cross cultural communication/  23. exp *cross cultural treatment/  24. ((cultur* or crosscultur* or intercultur* or transcultur* or multicultur*) adj3 (competenc* or understanding or knowledg* or expertise or skill* or sensitiv* or aware* or appropriate* or acceptab* or humility or service* or communicat* or barrier* or divers* or comparison* or identity or specific or background* or value* or belief* or literacy or respect or framework)).ti.  25. or/21-24  26. 5 and 20 and 25  27. 6 or 26 | |

4. ERIC

| Interface: ProQuest  Date of Search: 29 March 2019  Number of hits: 28 | Field labels   - MAINSUBJECT.EXACT.EXPLODE = exploded controlled term - MAINSUBJECT.EXACT = non exploded controlled term - ti = title - ab = abstract - NEAR/x = within x words, regardless of order - * = truncation of word for alternate endings |
| --- | --- |
| ((ti(((cultur* OR crosscultur* OR intercultur* OR transcultur* OR multicultur*) NEAR/3 (competenc* OR understanding OR knowledg* OR expertise OR skill* OR sensitiv* OR aware* OR appropriate* OR acceptab* OR humility OR service* OR communicat* OR barrier* OR divers* OR comparison* OR identity OR specific OR background* OR value* OR belief* OR literacy OR respect OR framework)) NEAR/3 (education* OR teaching OR learning OR elearning OR training OR curriculum OR "tacit knowledge" OR intervention* OR program*)) OR ab(((cultur* OR crosscultur* OR intercultur* OR transcultur* OR multicultur*) NEAR/3 (competenc* OR understanding OR knowledg* OR expertise OR skill* OR sensitiv* OR aware* OR appropriate* OR acceptab* OR humility OR service* OR communicat* OR barrier* OR divers* OR comparison* OR identity OR specific OR background* OR value* OR belief* OR literacy OR respect OR framework)) NEAR/3 (education* OR teaching OR learning OR elearning OR training OR curriculum OR "tacit knowledge" OR intervention* OR program*))) AND (MAINSUBJECT.EXACT("Physicians") OR MAINSUBJECT.EXACT("Family Practice (Medicine)") OR ti("general practi*" OR gp OR gps OR "family practi*" OR "primary health care" OR "primary healthcare" OR "primary care") OR ab("general practi*" OR gp OR gps OR "family practi*" OR "primary health care" OR "primary healthcare" OR "primary care"))) OR ((MAINSUBJECT.EXACT("Physicians") OR MAINSUBJECT.EXACT("Family Practice (Medicine)") OR ti("general practi*" OR gp OR gps OR "family practi*" OR "primary health care" OR "primary healthcare" OR "primary care") OR ab("general practi*" OR gp OR gps OR "family practi*" OR "primary health care" OR "primary healthcare" OR "primary care")) AND (MAINSUBJECT.EXACT("Graduate Medical Education") OR MAINSUBJECT.EXACT("Education") OR MAINSUBJECT.EXACT.EXPLODE("Educational Methods") OR MAINSUBJECT.EXACT("Teaching") OR MAINSUBJECT.EXACT.EXPLODE("Learning") OR MAINSUBJECT.EXACT.EXPLODE("Curriculum") OR MAINSUBJECT.EXACT("Hidden Curriculum") OR MAINSUBJECT.EXACT("Informal Education") OR ti(education* OR teaching OR learning OR elearning OR training OR curriculum OR "tacit knowledge" OR intervention* OR program*) OR ab(education* OR teaching OR learning OR elearning OR training OR curriculum OR "tacit knowledge" OR intervention* OR program*)) AND (MJMAINSUBJECT.EXACT("Cultural Literacy") OR MJMAINSUBJECT.EXACT("Cultural Awareness") OR MJMAINSUBJECT.EXACT("Cross Cultural Training") OR MJMAINSUBJECT.EXACT("Intercultural Communication") OR ti((cultur* or crosscultur* or intercultur* or transcultur* or multicultur*) NEAR/3 (competenc* or understanding or knowledg* or expertise or skill* or sensitiv* or aware* or appropriate* or acceptab* or humility or service* or communicat* or barrier* or divers* or comparison* or identity or specific or background* or value* or belief* or literacy or respect or framework)))) | |
