# Supplementary material for: The informal curriculum of family medicine – what does it entail and how is it taught to residents? A systematic review
Source: BMC Fam Pract. 2020 Mar 11;21:49. doi: 10.1186/s12875-020-01120-1 (PMC7066821; doi:10.1186/s12875-020-01120-1)
Supplement: Supplementary file 5 — Additional file 5. A summary of the studies included for question 1. Articles included are listed and described using the following headings: “defines informal curriculum”, “type of article”, “participants”, and “elements of the informal curriculum”. [file 12875_2020_1120_MOESM5_ESM.docx]

**Additional file 5. A summary of the studies included for question 1**

| First author  Year | Defines informal/ hidden curriculum | Type of article | Participants | Elements of the informal curriculum |
| --- | --- | --- | --- | --- |
| Culhane-Pera2000 (25) | Yes | Survey | 476 family practice residency programs | Cultural competence |
| Law  2016 (32) | No | Discussion | None | Professionalism |
| Paul  2019 (26) | Yes | Qualitative | 3 Family Medicine residents and  7 medical students | Cultural competence |
| Pimlott  2018 (29) | No | Editorial | None | Professionalism |
| Senior  2015 (28) | Yes | Discussion | None | Dealing with uncertainty  Cultural competence  Professionalism |
| Sturman  2012 (33) | No | Qualitative | 13 GP clinical teachers | Professionalism  Dealing with uncertainty |
| Watt  2015 (27) | Yes | Qualitative | 15 GP supervisors | Cultural competence |
| Watt  2016 (30) | No | Review | None | Cultural competence |
| Watt  2016 (31) | No | Survey | 43 GP registrars | Cultural competence |
