# Supplementary material for: The informal curriculum of family medicine – what does it entail and how is it taught to residents? A systematic review
Source: BMC Fam Pract. 2020 Mar 11;21:49. doi: 10.1186/s12875-020-01120-1 (PMC7066821; doi:10.1186/s12875-020-01120-1)
Supplement: Supplementary file 6 — Additional file 6. A summary of the studies included for question 2. Articles included are listed and described using the following headings: “informal curriculum theme”, “evaluation methods”, “expected learning outcome”, “context”, “impact of intervention”, and “strength of findings”. [file 12875_2020_1120_MOESM6_ESM.docx]

**Additional file 6. A summary of the studies included for question 2**

| First author  Year | Informal curriculum theme | Evaluation methods | Expected learning outcome | Context | Impact of intervention  (Kirkpatrick) | Strength of findings  (BEME) |
| --- | --- | --- | --- | --- | --- | --- |
| Chen  2017 (17) | Professionalism | Design: Qualitative  Data collection:  Semi-structured interviews | Improved professional identity/role understanding | No of subjects: 31  Country of study: Canada  Duration/frequency of exposure: Academic half-days weekly-monthly over one year.  Setting/intervention: Classroom teaching. Peer-support and mentorship | 2 | 2 |
| Clanidin 2008 (53) | Professionalism | Design: Narrative reflective approach  Data collection: Charts written by participants | Improved professional development | No of subjects: 4  Country of study: Canada  Duration/frequency of exposure: Weekly sessions during 10 weeks  Setting/intervention: Reflecting on charts in small-group discussions | 2 | 1 |
| Rutherford 2018 (51) | Professionalism | Design: Mixed methods. Non-comparative  Data collection: Photographs and reflective writing analyzed qualitatively. Questionnaire | Enhanced reflective capacity, self-awareness and professional development | No of subjects: 8  Country of study: UK  Duration/frequency of exposure: 2 sessions  Setting/intervention: Small-group workshops and reflective writing | 2 | 2 |
| Pavlov  2010 (52) | Professionalism | Design: Not clearly stated  Data collection: Not clearly stated | Improved communication skills and professional behavior | No of subjects: 9  Country of study: USA  Duration/frequency of exposure: 1 session  Setting/intervention: Small group discussion/reflection on clips from Grey’s anatomy | 2 | 1 |
| Player  2018 (50) | Professionalism | Design: Qualitative  Data collection:  Semi-structured interviews | Improved understanding and skilful management of doctor–patient relationships | No of subjects: 18  Country of study: USA  Duration/frequency of exposure: Session twice monthly for one year  Setting/intervention: Participation in Balint seminars (small group settings) | 2 | 3 |
| Chudley 2007 (49) | Cultural competence | Design: Quantitative. Single group comparative pre-post  Data collection: Pre-post questionnaire | Improved cultural competence with focus on consultation skills | No of subjects: 173  Country of study: UK  Duration/frequency of exposure: 1-day course  Setting/intervention: Small group discussion and role play | 2 | 3 |
| Duncan  2007 (47) | Cultural competence | Design: Quantitative. Non-comparative study  Data collection:  Observations and questionnaire | Improved cultural competence (primarily with respect to Australian culture and language) | No of subjects: 16  Country of study: Australia  Duration/frequency of exposure:  A number of workshops – unclear how many  Setting/intervention: Small-group sessions during which poetry was discussed and role plays enacted | 2 | 1 |
| Green  2008 (46) | Cultural competence | Design:  Quantitative. Two group comparative (intervention/control)  Data collection:  Questionnaire | Improved cultural competence | No of subjects: 1467  Country of study: USA  Duration/frequency of exposure: One year, i.e. last year of residency training  Setting/intervention: A specially designed program involving clinical immersion in diverse communities in parallel with small-group discussions, lectures and seminars | 2 | 3 |
| Juarez  2006 (48) | Cultural humility (competence) | Design:  Quantitative. Single-group comparative pre-post  Data collection: Interviews, ratings by patients and observational data | Improved cultural humility (competence) | No of subjects: 11  Country of study: USA  Duration/frequency of exposure: Monthly sessions during one year  Setting/intervention:  Lectures, simulated patients, clinical immersion, book discussions, panel discussion, interview training | 3 | 3 |
| Kutob  2009 (45) | Cultural competence | Design:  Quantitative. RCT  Data collection:  Pre-post questionnaire | Improved cultural competence in the context of type 2 diabetes. | No of subjects: 122  Country of study: USA  Duration/frequency of exposure: One session  Setting/intervention: Internet-based course on cultural competence in diabetes care | 2 | 3 |
| Taylor 2018 (44) | Uncertainty | Design: Quantitative. Single-group comparative pre-post  Data collection:  Pre-post questionnaire (four different surveys) | Increased tolerance of ambiguity | No of subjects: 25  Country of study: USA  Duration/frequency of exposure: A curriculum encompassing a yearly 4-week outpatient family medicine-teaching rotations each year of residency training  Setting/intervention: Clinical immersion. Reflective writing and discussion. Each participant spent 50% of their time in the clinic and 50% in formal educational sessions. | 2 | 2 |
