# Supplementary material for: The informal curriculum of family medicine – what does it entail and how is it taught to residents? A systematic review
Source: BMC Fam Pract. 2020 Mar 11;21:49. doi: 10.1186/s12875-020-01120-1 (PMC7066821; doi:10.1186/s12875-020-01120-1)
Supplement: Supplementary file 7 — Additional file 7. A summary of the development of concepts and themes from articles included for question 1. Summarizes data underlying the development of the concepts and themes for question 1. [file 12875_2020_1120_MOESM7_ESM.docx]

**Additional file 7. A summary of the development of concepts and themes from articles included for question 1**

| REFERENCE | DEFINTION OF INFORMAL/HIDDEN CURRICULUM | EDUCATIONAL ELEMENTS | CONCEPTS | THEMES Of THE INFORMAL/HIDDEN CURRICULUM |
| --- | --- | --- | --- | --- |
| Culhane-Pera 2000, Multicultural curricula in family practice residencies. | Informal curriculum was defined in the survey as, *“The topic has been included in the residency but has not been organized into a coherent whole with*  *written goals, objectives, methods, and/or evaluation.”*  Formal curriculum was defined in the survey as, *“The topic has been organized into a coherent whole and has been written into curricular components such as goals, objectives, methods, and/or evaluation.”* | Cultural competence discussed in terms of: *“an understanding of patients’ cultural beliefs, values, and health care practices”*,  outlined by six content areas: *“influence of cultural issues in clinical settings, general cultural issues, self-awareness, epidemiology, biomedicine, and complementary medicine.”*  *“Eighty-six percent of all responding residencies reported teaching about multicultural issues in health care; 58% had an informal curriculum, 28% had a formal program, and 14% had no curriculum.”*  *“Programs with*  *formal curricula reported teaching significantly more*  *content areas with a greater variety of methods, used*  *more evaluation methods, and evaluated themselves as*  *more successful than programs with informal curricula.”* | Identifies cultural competence as being part of an informal curriculum in the majority of family medicine residency programs | Gaining cultural competence |
| **Law 2016,** Deconstructing “Mabel” | None provided, but mentioned as important: *“Informal and hidden curricula play a substantial role in fostering professionalism”.* | *“Informal and hidden curricula play a substantial role in fostering professionalism and a primary source of this learning is role modelling.”* | Professionalism learned through role modelling as part of informal/hidden curricula | Achieving medical professionalism |
| **Paul 2019,** Lining up the ducks: Aligning the formal, informal and hidden curricula in an immersed learning environment | The hidden curriculum includes *“a set of influences that function at the level of*  *organizational structure and culture” (Hafferty, 1998, p. 404). It is the space where a student’s tacit inculcation into the culture of medicine occurs, through “the unstated, taken-for-granted, and sometimes unconscious group understandings about how things are done within the group” (O'Donnell, 2015, p. 7)*  Informal and formal curriculum not defined. | *“Through their placements, they gained experiential knowledge around the relevance of a holistic approach”*  *“Specifically, participants understood the interconnectedness of health, family and community for Aboriginal people”*  *“Participants gained an understanding of how exercising patience and being non-judgemental was crucial for developing practitioner–patient trust and rapport.”*  *“Participants revealed an increased appreciation of the vital nature of prioritising the*  *development of a relationship with patients”*  *“Participants in our study became aware of differences, self and power in their relationships*  *with their patients—a care approach that is imperative to culturally-safe cross-cultural practitioner–patient relationships”* | Importance of holistic approach in a culturally safe healthcare  Importance of building a sound patient-doctor relationship  Importance of self-awareness and reflection | Gaining cultural competence  Achieving medical professionalism |
| **Pimlott, 2018**  The hidden curriculum and continuing professional development for family physicians | None.  Discussed in terms of: pervading undergraduate and postgraduate medical training  *“…hidden curriculum extends well beyond medical school and into our lifelong continuing professional development...”* | *“…the “hidden curriculum,” which pervades undergraduate and postgraduate medical training, promotes a hierarchy between generalists and specialists in which the generalist is portrayed as inferior”*  *“For specialists it will mean relinquishing their hold on the “evidence,” working in truly collaborative ways with family physicians, and not defending the status quo.* | Not reinforcing a hierarchy between generalists and specialists  Working collaboratively (specialists and generalists) – not defending status quo | Achieving medical professionalism |
| **Senior 2015,**  Writing the hidden curriculum | *“The hidden curriculum is the set of values that students learn from, no matter what we have decided to teach them.”* | *“There are other things I’d put in my written- down hidden curriculum; routinely basing case scenarios on patients from different class and cultural backgrounds so we avoid stereotyping white and middle-class patients as ‘normal’.*  *“…watch and learn*  *from a whole range of doctors, something*  *often denied to us later in our careers.*  *This exerts a powerful influence over what*  *behaviour is acceptable as a doctor”*  *“Part of my hidden*  *curriculum will routinely ask patients for advice for myself […] inviting a sharing of expertise”*  *“Sometimes we see people with problems*  *that don’t have a medical diagnosis or a*  *medical solution. But never do we see case-*  *scenarios without a diagnosis or solution.*  *Every problem we have ever been taught*  *about has an answer that is correct, and*  *there will be an authority somewhere to tell*  *us what the answer is.”*  *“My proposed hidden curriculum would*  *suggest that teachers show themselves*  *doing strange things normally kept out of*  *sight, like being human. Revealing what happens when we don’t know something”* | Case-based education should reflect a culturally diverse population  Acceptable professional behaviour identified through observation  Engaging the patient in establishing a good patient-doctor relationship  Dealing with not knowing the answer/when there is no correct answer | Gaining cultural competence  Achieving medical professionalism  Dealing with uncertainty |
| **Sturman 2012,** The informal curriculum – GPs perception of ethics in clinical practice | No clear definition but uses term informal curriculum in relation to inter-personal learning : *“The informal curriculum of inter-personal*  *learning in clinical environments is likely to be more important in the process of moral enculturation of students than formally structured medical student teaching in the domain of ethics.”* | Informal curriculum of “ethical issues” in general practice include (according to the authors):   - Patient-doctor relationships - Professional differences   *“covering for a colleague” “inter-professional tensions” “respect for other therapeutic clinical relationships”*   - Truth telling – e.g. managing uncertainty about patient truthfulness - Ethically “grey” areas – dealing with ethical uncertainty or ambivalence - . *“Participants*   *perceived ‘grey’ areas and challenges, rather than*  *‘black and white’ ethical lapses or contradictions*  *between formal teaching and actual practice”*   - The personal demands of ethical decision making e.g. “*personal effort*   *involved in denying inappropriate patient requests”; ” costs included personal vulnerability to clinical diagnosis (e.g. a missed diagnosis)*  *“There is some evidence that the ability of medical students to recognise, and respond to, professional lapses and ethically problematic behaviour decreases over their clinical years as a result of their experience of learning in*  *‘real world’ healthcare settings, where they*  *may see professional role models behaving in*  *ways they perceive to be unethical. This has been described as an ‘ethical erosion’ of medical*  *student moral sensitivity. This informal curriculum*  *has been explored through thematic analyses of*  *reflective student essays about their experiences,*  *predominantly in hospital settings.”* | Importance of building a sound patient-doctor relationship  Maintaining sound relationships with colleagues  Dealing with uncertainty in relation to what to discuss overtly with patients  Dealing with uncertainty in terms of “appropriate” behaviour  Dealing with uncertainty inherent in medicine and in relation terms of "appropriate” behaviour  Acceptable professional behaviour identified through observation | Achieving medical professionalism  Dealing with uncertainty  Achieving medical professionalism |
| **Watt 2015,**  Cultural competency training of GP Registrars-exploring the views of GP Supervisors | *“The influence that institutional culture and structure play in*  *learning, (‘the hidden curriculum’), has been noted to*  *affect outcomes of training with respect to cultural competency.”* | *“Many supervisors viewed the development of knowledge of culturally sensitive topics, historical factors and health beliefs as best gained through the experience of working day-to-day* *as a general practitioner.”*  *“Cross-cultural aspects of care addressed during supervision occurred mostly on an ad hoc basis, in response to registrar difficulties with particular patient encounters or as a result of patient feedback where cultural differences were perceived by the registrar or supervisor*  *to be impacting.”*  *“Formal training for both registrars and supervisors may be beneficial, not only to develop a deeper understanding of cultural competence and its relevance to practice, but*  *also to promote more consistency in learning.”* | Cultural competence is currently gained mainly through experience  Cross-cultural aspects of care addressed on ad hoc basis  Formal training in cultural competence may be beneficial | Gaining cultural competence |
| **Watt, 2016**  Developing cultural competence in general practitioners: an integrative review of the literature | No clear definition.  Does not explicitly use the terms hidden or informal curriculum but refers to informal learning. | *“Formal cultural competence training in General Practice appeared to be underdeveloped despite GP registrars generally desiring more training. The development of most aspects of cultural competence relied on informal learning and in-practice exposure but this required proper guidance and facilitation by supervisors and educators.”*  *The amount of formal*  *training appears to increase GP registrars’ preparedness*  *and competence to provide cross-cultural care more so*  *than having good role models or exposure to greater diversity cross-cultural case mix throughout training [35].*  *“The literature suggests that much of the development of cultural competence in General Practice occurs informally, as fits with the work-place based training common to most GP programs. However, best practice delivery of cultural competence training in this setting has not been well explored.”* | The development of cultural competence relies on informal learning  Formal training in cultural competence may be beneficial  Most GP programs are based on work-place based training, in which learning/developing of competencies e.g. cultural competence occurs informally |  |
| **Watt 2016,**  Cross-cultural training of general practitioner registrars: how does it happen? | Not defined.  Informal learning/opportunistic learning/ad hoc learning are all terms mentioned in relation to cross-cultural training | *“the majority of registrars believed it was an appropriate role for the GP supervisor to train them in cultural competence.”*  *“I guess we don’t receive any cultural training. It´s all about what we discover on day-to-day basis.”*  *“Exposure to cultural diversity is an important method of developing cultural competence (…) However, exposure alone does not guarantee cultural competence.”*  *“…relying on individual patient encounters as the triggers for learning cultural competence depends somewhat on chance, and is influenced by the supervisor’s interest and ability to bring focus to the topic.”*  *“…cross-cultural learning mainly occurred informally rather than through learning opportunities focused on specific cultural competence skills development and cultural self-reflection.”* | Cross-cultural learning dependent on supervisor  Cross-cultural learning mainly occurs informally  Exposure to cultural diversity is important, but does not suffice on its own for gaining cultural competence  Learning cultural competence by chance  Cross-cultural learning mainly occurs informally | Gaining cultural competence |
